# A New Synthetic Compound, 2-OH, Enhances Interleukin-2 and Interferon-γ Gene Expression in Human Peripheral Blood Mononuclear Cells

**DOI:** 10.3390/molecules14072345

**Published:** 2009-07-02

**Authors:** Shiu-Huey Chou, Shang-Shing P. Chou, Yih-Fong Liew, Jyh-Yih Leu, Su-Jane Wang, Rwei-Fen S. Huang, Woan-Fang Tzeng, Yuh-Chi Kuo

**Affiliations:** 1 Department of Life Science, Fu-Jen University, No. 510, Chung-Cheng Rd., Hsinchuang, Taipei Hsien 242, Taiwan; E-mails: 047751@mail.fju.edu.tw (S-H.C), 049432@mail.fju.edu.tw (J-Y.L.), 012098@mail.fju.edu.tw (W-F.T.); 2 Department of Chemistry, Fu-Jen University, No. 510, Chung-Cheng Rd., Hsinchuang, Taipei Hsien 242, Taiwan; E-mail: chem1004@mail.fju.edu.tw (S-S.P.C.); 3 Department of Nutritional Science, Fu-Jen University, No. 510, Chung-Cheng Rd., Hsinchuang, Taipei Hsien 242, Taiwan; E-mails: 070647@mail.fju.edu.tw (Y-F.L.), rweifen@mail.fju.edu.tw (R-F.H.); 4 School of Medicine, Fu-Jen University, No. 510, Chung-Cheng Rd., Hsinchuang, Taipei Hsien 242, Taiwan; E-mail: med0003@mail.fju.edu.tw (S-J.W.)

**Keywords:** 2-OH, PBMC, proliferation, IL-2

## Abstract

A new synthetic compound, 6-hydroxy-2-tosylisoquinolin-1(2*H*)-one (**2-OH**), was selected for immunopharmacological activity tests. The effects of **2-OH** on human peripheral blood mononuclear cell (PBMC) proliferation were determined by tritiated thymidine uptake. Compared to phytohemagglutinin (PHA; 5 μg/mL) stimulation, **2-OH** significantly enhanced PBMC proliferation in a dose-dependent manner. The 50% enhancement activity (EC_50_) for **2-OH** was 4.4±0.1 μM. In addition, effects of **2-OH** on interleukin-2 (IL-2) and interferon-γ (IFN-γ) production in PBMC were determined by enzyme immunoassay. Results demonstrated that **2-OH** stimulated IL-2 and IFN-γ production in PBMC. Data from reverse transcription-polymerase chain reaction (RT-PCR) and real-time PCR indicated that IL-2 and IFN-γ mRNA expression in PBMC could be induced by **2-OH**. Therefore, **2-OH** enhanced IL-2 and IFN-γ production in PBMC by modulation their gene expression. We suggest that **2-OH** may be an immunomodulatory agent.

## 1. Introduction

Many bioactive compounds have a piperidine structure [1]. For example, we have proved that (*S*)-armepavine (C_19_H_23_NO_3_) from *Nelumbo nucifera* inhibits the proliferation of human blood mononuclear cells (PBMC) activated with phytohemagglutinin (PHA) by regulation of PI3K activation [2,3]. (*S*)-Armepavine also improves autoimmune diseases in MRL/MpJ-*lpr/lpr* mice [4]. Bortezomib (C_19_H_25_BN_4_O_4_) is a proteasome inhibitor used for treatment multiple myeloma [5]. Recently, a new piperidine compound, 6-hydroxy-2-tosylisoquinolin-1(2*H*)-one (**2-OH**) has been synthesized in the laboratory of co-author Chou [6], but there has been relatively scarce definitive evidence to prove its pharmacological activities. Herein, we investigate the immunomodulatory functions of **2-OH**.

The central event in generation of immune responses is the activation and clonal expansion of T cells [7]. Interaction of T cells with antigens initiates a cascade of biochemical events and gene expression that induces resting T cells to proliferate and differentiate [8]. It has been demonstrated in many previous studies with T cells that a series of genes such as interleukin-2 (IL-2) and interferon-γ  (IFN-γ) are pivotal in the growth of T lymphocytes induced by antigens [9,10]. Thus, growth modulators or other external events that affect the T cell proliferation are likely to act by controlling the expression or function of the products of these genes [11]. Regulation of T lymphocyte activation and proliferation and cytokine production has been shown to be one of actions of immunomodulatory drugs [12,13].

In order to prove the immunomodulatory effects of **2-OH**, human peripheral blood mononuclear cells (PBMC), which contain T lymphocytes, were used as target cells [14,15]. Phytohemagglutinin (PHA) is a mitogen for T lymphocytes. It binds to *N*-acetylgalactosamine glycoproteins expressed on the surface of T cells, then activates the cells to proliferate and is applied as a positive control [16]. To elucidate the effects of **2-OH** on PBMC proliferation, the tritiated thymidine uptake method was utilized to detect total cellular DNA synthesis in the cultures. In addition, we determined the actions of **2-OH** on production and gene expression of IL-2 and IFN-γ in PBMC by enzyme immunoassay (EIA) and reverse transcription-polymerase chain reaction (RT-PCR), respectively, and examined regulatory roles of **2-OH** on PBMC activation and proliferation.

## 2. Results and Discussion

**Figure 1:**
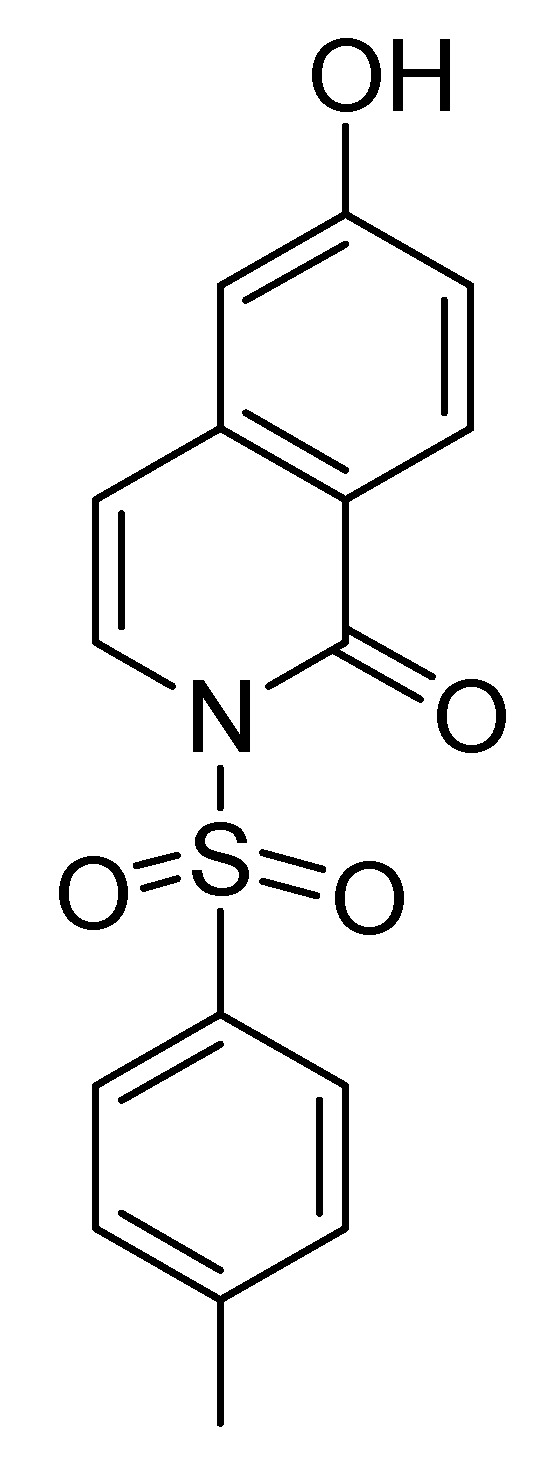
The structure of **2-OH** (C_16_H_13_NO_4_S; M.W. 315).

**Figure 2:**
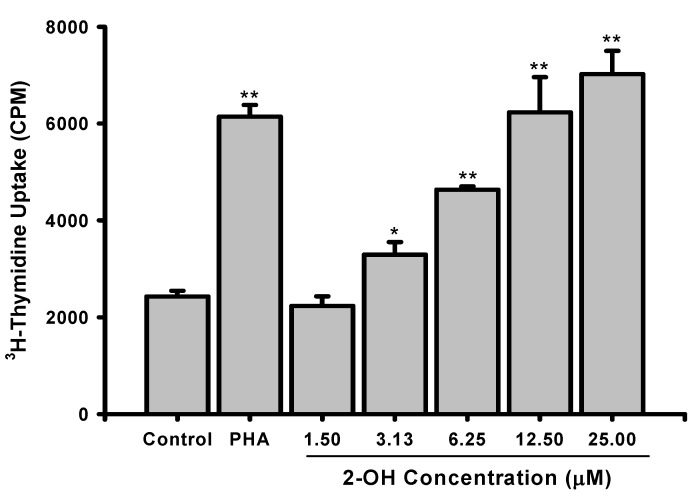
Effects of **2-OH** on PBMC proliferation. PBMC (2×10^5^/well) were treated with 0.1% DMSO (control), PHA (5 µg/mL) or indicated concentrations of **2-OH** (1.5, 3.13, 6.25, 12.5, and 25 μM) for three days. The proliferation of cells were detected by tritiated thymidine uptake. After a 16 hr incubation, the cells were harvested by an automatic harvester then radioactivity was measured by a scintillation counting. Each bar represents the mean±S.D. of three independent experiments with PBMC from different individuals. *P < 0.05, **P< 0.01, as compared with control group.

The structure of **2-OH** is shown in Figure 1. To study the effects on PBMC cell proliferation, the cells were treated with PHA (5 μg/mL) or various concentrations of **2-OH** (1.5 to 25 μM) and cell proliferation was determined by tritiated thymidine uptake. As shown in Figure 2, treatment with PHA for three days stimulated cell proliferation by about 2.5 fold, as reflected by the increase in tritiated thymidine uptake (2,429±118 vs. 6,142±243 CPM, P < 0.01). The enhancement effects of **2-OH** were concentration dependent and its activity was compatible with PHA. At 6.25 μM, the percentage enhancement of **2-OH** is 91±3% (P<0.01). The corresponding degree of enhancement for 25 μM is 189±20% (P<0.01). Moreover, 50% enhancement activity (EC_50_) of **2-OH** on PBMC proliferation is 4.4±0.1 μM. PBMC proliferation was not changed by the vehicle (control; 0.1% DMSO) because the data showed there was no difference in cell proliferation between medium and vehicle groups (2,210±189 vs. 2,429±118 CPM). Therefore, the enhancement observed with **2-OH** was unlikely related to DMSO. These data indicated that **2-OH** exhibited inductive activity on PBMC proliferation.

**Figure 3:**
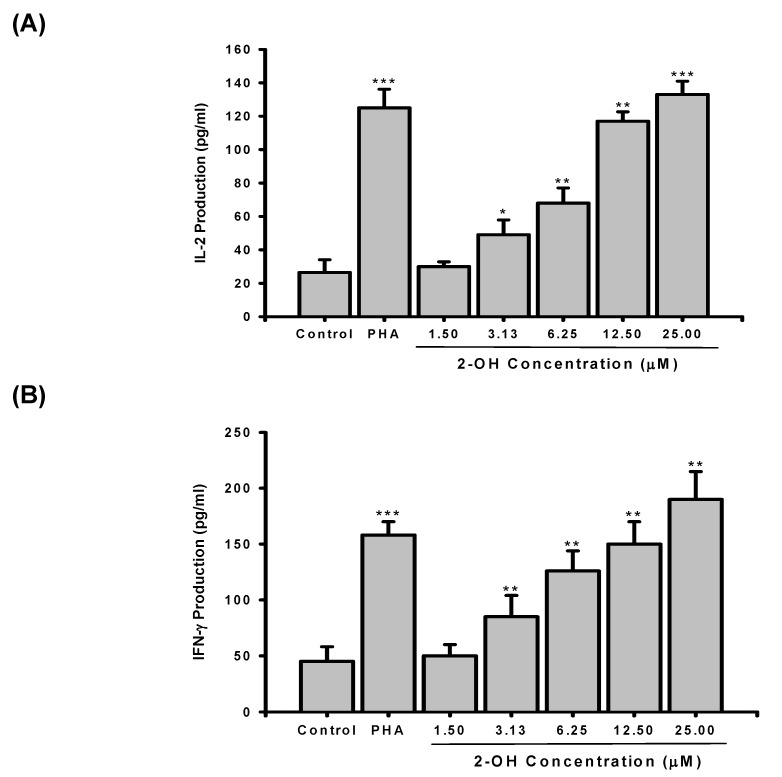
The IL-2 and IFN-γ production in PBMC cultures treated with **2-OH**. PBMC (2×10^5^/well) were treated by 0.1% DMSO (control), PHA (5 µg/mL) or indicated concentrations of **2-OH** (1.5, 3.13, 6.25, 12.5, and 25 μM) for three days. Then the cell supernatants were collected and (A) IL-2 and (B) IFN-γ concentrations were determined by EIA. Each bar is the mean±S.D. of three independent experiments with PBMC from different individuals. *P < 0.05, **P< 0.01, ***P< 0.001, as compared with control group.

To study whether enhancement of PBMC proliferation was related to production of cytokines, the cells were incubated for three days with or without various concentration of **2-OH** (1.5 to 25 μM). Supernatants were then collected, and the production of IL-2 and IFN-γ assayed by EIA. As shown in Figure 3A, the stimulated production of IL-2 in PBMC was significantly enhanced by PHA (26.5±7.6 vs. 125±11.2 pg/mL, P < 0.001). Furthermore, IL-2 production in PBMC was enhanced by **2-OH**. At 1.5 to 25 μM, the percentages of enhancement of IL-2 production by **2-OH** were 13±11%, 85±34%, 157±34%, 341±20%, and 402±30%, respectively. As shown in Figure 3B, treatment with PHA for three days stimulated cell production of IFN-γ by about 3.5 fold (45±13 vs. 158±12 pg/mL, P < 0.001). **2-OH** stimulates IFN-γ production in PBMC with a dosage-dependent manner. From 3.13 to 25 μM, **2-OH** significantly increased IFN-γ production in PBMC (P < 0.01). These results demonstrated that **2-OH** was similar to PHA to induce IL-2 and IFN-γ productions in PBMC.

In the present study, we also proved that 0.1% DMSO (control group) did not cause PBMC to produce IL-2 (29.8±9.5 vs. 26.5±7.6 pg/mL) and IFN-γ  (40.8±15 vs. 45±13 pg/mL), as compared with medium, so we suggest that **2-OH** enhancement of cytokine production is not due to DMSO. Many studies have indicated that production of cytokines such as IL-2 and IFN-γ are involved in regulation of PBMC proliferation. The agents that affect PBMC proliferation are ultimately likely to control the expression or function of IL-2 and IFN-γ [17,18]. In this study, we proved that **2-OH** enhanced proliferation and IL-2 and IFN-γ production in PBMC. Thus, we predict that one of factors contributing to the stimulation of PBMC proliferation by **2-OH** is an increase in IL-2 and IFN-γ production.

To demonstrate whether **2-OH** enhanced IL-2 and IFN-γ production in PBMC through induction of mRNA transcripts, total cellular RNA was extracted from PBMC in the presence or absence of 6.25 μM and 25 μM **2-OH** and used for RT-PCR. The oligonucleotide sequences of IL-2, IFN-γ, and glyceraldehyde-3-phosphate dehydrogenase (GAPDH) primers are shown in Table 1. 

**Table 1:** Oligonucleotide sequences of the primers used for amplification of IL-2 and IFN-γ in PBMC.

Cytokine	Sequence	Predicted size (bp)
IL-2	5’-GTC ACA AAC AGT GCA CCT AC-3’ 5’-GAA AGT GAA TTC TGG GTC CC-3’	262
IFN-γ	5’-GCA GAG CCA AAT TGT CTC CT-3’ 5’-ATG CTC TTC GAC CTC GAA AC-3’	320
GAPDH	5’- TGA AGG TCG GAG TCA ACG GAT TTG GT-3’ 5’- CAT GTG GGC CAT GAG GTC CAC CAC-3’	983

**Figure 4:**
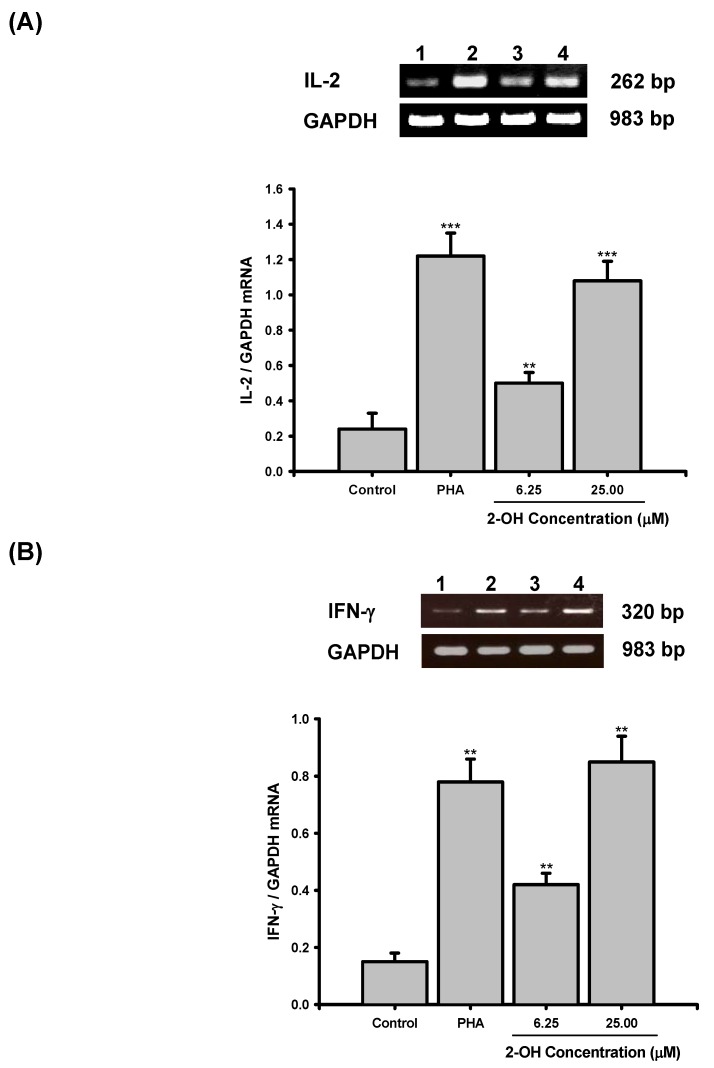
Effects of **2-OH** on: (A) IL-2 and (B) IFN-γ mRNA expression in PBMC, as detected with RT-PCR. PBMC (5 x 10^6^) were cultured with PHA (5 µg/mL) or 6.25 and 25 μM **2-OH** for 18 hr. The total cellular RNA was isolated from PBMC and aliquots of RNA (1 μg) were reverse-transcribed for synthesis of cDNA. PCR was done as described in Materials and Methods. Following the reaction, the amplified product was taken out of the tubes and run on 2% agarose gel. Lane 1: Control (0.1% DMSO), Lane 2: PHA, Lane 3: 6.25 μM **2-OH**, Lane 4: 12.5 μM **2-OH**. Each band was quantitated using laser scanning densitometer SLR-2D/1D (Biomed Instruments Inc., Fullerton, CA, USA). The ratio of IL-2 or IFN-γ mRNA to GAPDH mRNA was calculated. Each bar is the mean±S.D. of three independent experiments with PBMC from different individuals. **P < 0.01, ***P < 0.001, as compared with control group.

The results of RT-PCR analyses are shown in Figure 4. As shown in Figure 4A and Figure 4B, the mRNA for  GAPDH was detectable in the samples treated with vehicle (control; 0.1% DMSO; Lane 1), PHA (Lane 2), 6.25 μM **2-OH** (Lane 3), and 25 μM **2-OH** (Lane 4), respectively, and neither 6.25 μM nor 25 μM **2-OH** affected GAPDH mRNA expression in PBMC. Whereas unstimulated PBMC (control group) expressed a little IL-2 and IFN-γ  mRNAs, the levels of both cytokine mRNAs were significantly increased in PBMC activated with PHA (IL-2, P < 0.001; IFN-γ, P < 0.01). Compared with the control group, PCR products for the IL-2 and IFN-γ mRNAs amplified from PBMC RNA preparations were increased by 6.25 μM and 25 μM **2-OH**. Laser densitometry analysis demonstrated that the ratios of IL-2  to GAPDH mRNA in PBMC were significantly increased by 6.25 μM (P < 0.01) and 25 μM (P < 0.001) **2-OH** (Figure 4A). As shown in Figure 4B, the ratios of IFN-γ to GAPDH mRNA were also significantly induced by 6.25 μM (P < 0.01) and 25 μM (P < 0.01) **2-OH**. 

The enhancement actions of **2-OH** on IL-2 and IFN-γ mRNA expression were also confirmed by the real-time PCR (Table 2). The positive control PHA significantly increased IL-2 (P < 0.01) and IFN-γ (P < 0.05) transcripts in PBMC. Comparison with the control group, **2-OH** significantly decreased ΔC_T_ values of IL-2 (6.25 μM, P < 0.05; 25 μM, P < 0.001) and IFN-γ (6.25 μM, P < 0.05; 25 μM, P < 0.01) in PBMC.

The RT-PCR and real-time PCR results proved that the levels of IL-2 and IFN-γ transcripts could be enhanced in **2-OH**-treated PBMC. By real-time PCR, we have demonstrated that 0.1% DMSO did not affect IL-2 (8.75±0.22 vs. 8.96±0.10 ΔC_T_) and IFN-γ  (9.38±0.92 vs. 9.62±0.87 ΔC_T_) gene expression in PBMC. It suggests that the increases in IL-2 and IFN-γ transcripts in the presence of **2-OH** are not related to vehicle. In the present study, production of cytokines such as IL-2 and IFN-γ  in PBMC cultures were increased by **2-OH**. We concluded that the enhancements of IL-2 and IFN-γ productions in PBMC were related to **2-OH** stimulating the mRNA transcription of these cytokines. Furthermore, calcium is a major secondary messenger that is activated during cell proliferation [7,17]. The decreasing of intracellular Ca^2+^ concentration would induce impairments of IL-2 and IFN-γ transcripts in PBMC [13]. Recently, we have proved that an analogue of **2-OH**, 3,4,4a,5,8,8a-hexahydro-6,7-dimethyl-4a-(phenylsulfonyl)-2-tosylisoquinolin-1(2*H*)-one, has the ability to regulate Ca^2+^ influx into nerve cells [19]. We suggest that induction of IL-2 and IFN-γ gene expression by **2-OH** may be through the modulation of Ca^2+^ mobilization.

**Table 2:** ΔC_T_ for IL-2 and IFN-γ  at 2-OH-treated PBMC.

	C_T_			IL-2 ΔC_T_	IFN-γ ΔC_T_
	IL-2	IFN-γ	GAPDH		
Control	34.34±0.69	35.05±0.15	25.38±0.79	8.96±0.10	9.62±0.87
PHA (5 μg/mL)	27.97±0.34	28.25±0.19	24.57±0.04	3.40±0.38**	3.76±0.11*
2-OH (6.25 μM)	30.56±0.45	31.27±0.04	24.87±0.11	5.69±0.56*	6.40±0.16*
2-OH (25 μM)	34.34±0.69	28.60±0.64	25.30±0.46	4.49±0.12***	3.33±0.20**

PBMC (5 × 10^6^ cells) were cultured with vehicle (control; 0.1% DMSO), PHA (5 μg/mL) or 6.25 and 25 μM **2-OH** for 18 hr. The cDNA was reverse-transcribed from cellular RNA and amplified by TaqMan PCR assay with an ABI prism 7700 sequence detection system. Each ΔC_T_ was calculated by subtracting the C_T_ of GAPDH mRNA from the C_T_ of IL-2 or IFN-γ mRNA, respectively. The data is the mean±S.D. from three independent experiments with PBMC from different individuals. *P < 0.05; **P < 0.01; ***P < 0.001, as compared with the control group.

## 3. Experimental

### 3.1. Preparation of 2-OH

The compound **2-OH** was prepared and characterized according to the method of Chou *et al.* [6]. Briefly, a toluene solution of 4-(phenylsulfonyl)-1-tosylpyridin-2(1H)-one and 1-methoxy-3-(trimethyl-silyloxy)-1,3-butadiene was heated in a sealed tube at 140 ^o^C for 24 h. The **2-OH** was dissolved in dimethylsulfoxide (DMSO) to a concentration of 100 mM and then stored at 4 ºC for use.

### 3.2. Preparation of PBMC

Twenty-two healthy male subjects (26 to 36 yr, mean age 29 yr) were chosen for this investigation. The experimental protocol had been reviewed and approved by the institutional human experimentation committee. Written informed consent was obtained from each and every subject. Heparinized human peripheral blood (35 ml) was obtained from healthy donors. PBMC was isolated by the Ficoll-Paque (specific gravity 1.077) gradient density method as described previously [20]. Thirty five mL of peripheral blood was centrifuged at 850 x g, 4 ºC for 10 min to remove the plasma. Blood cells were diluted with phosphate-buffered saline (PBS; pH7.2) then centrifuged in a Ficoll-Paque discontinuous gradient at 420 x g for 30 min. The PBMC layer was collected, washed with nine volumes of cold distilled water for 15 sec, and restored to normal tonicity by adding one volume of 10X Hanks' buffer saline solution (HBSS) to remove red blood cells. The cells were resuspended to a concentration of 2×10^6 ^cells/mL in RPMI-1640 medium supplemented with 2% fetal calf serum (FCS), 100 U/mL penicillin, and 100 µg/mL streptomycin. 

### 3.3. Lymphoproliferation test

The lymphoproliferation test was modified from a previously described procedure [21]. The density of PBMC was adjusted to 2×10^6 ^cells/mL before use. One hundred µL of cell suspension was applied into each well of a 96-well flat-bottomed plate (Nunc 167008, Nunclon, Raskilde, Denmark). PHA (Sigma) was used as a positive control [16]. PHA (5 μg/mL) or varying concentrations of **2-OH** (1.5 to 25 μM) were added to the cells. The plates were incubated in 5 % CO_2_-air humidified atmosphere at 37 °C for three days. Subsequently, tritiated thymidine (1 µCi/well, NEN) was added into each well. After incubation for 16 hr, the cells were harvested on glass fiber filters by an automatic harvester (Dynatech, Multimash 2000, Billingshurst, U.K.). Radioactivity in the filters was measured by a scintillation counting. The activity of 2-OH on PBMC proliferation was calculated by the following formula:

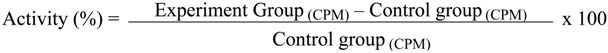


### 3.4. Determination of IL-2 and IFN-γ production

PBMC (2 × 10^5 ^cells/well) were cultured with PHA (5 μg/mL) or varying concentrations of 2-OH (1.5 to 25 μM) for 3 days. The cell supernatants were then collected and assayed for IL-2 and IFN-γ concentrations by EIA (R&D systems, Minneapolis, USA).

### 3.5. Extraction of total cellular RNA

PBMC (5 x 10^6^) were cultures with PHA (5 μg/mL) or **2-OH** (6.25 and 25 μM). PBMC were collected and lysed by RNA-Bee^TM^ (Tel-Test Inc., Friendswood, USA). After centrifugation, the supernatants were extracted with a phenol-chloroform mixture. The extracted RNA was precipitated with 100% cold ethanol. The total cellular RNA was pelleted by centrifugation and redissolved in diethyl pyrocarbonate (DEPC)-treated H2O. The concentration of RNA was calculated by measuring the optical density at 260 nm.

### 3.6. RT-PCR

The RT-PCR was performed by a method described previously [22]. Aliquots of 1 μg of RNA were reverse-transcribed to cDNA using the Advantage^TM^ RT-for-PCR kit from CLONTECH according to the manufacturer’s instructions. Briefly, 10 μL of cDNA was mixed with 0.75 μM primers, four units of Taq polymerase, 10 μL of reaction buffer (2 mM Tris-HCl, pH8.0; 0.01 mM ethylenediaminetetraacetate, EDTA; 0.1 mM dithiothreitol, DTT; 0.1% Triton X-100; 5% glycerol; and 1.5 mM MgCl_2_), and 25 μL of water in a total volume of 50 μL. All primer pairs for the IL-2, IFN-γ, and GAPDH were designed from the published human cDNA sequence data [23,24]. The PCR was done by the following setting of the air thermocycler: denaturing temperature of 94 °C for 1 min, annealing temperature of 60 °C for 1 min, and elongation temperature of 72 °C for 80 sec for the first 35 cycles and finally elongation temperature of 72 °C for 10 min. Following the reaction, the amplified products were taken out of the tubes and run on 2% agarose gel. 

### 3.7. Real-time PCR

The real-time PCR was performed by TaqMan PCR assay using an ABI prism 7700 sequence detection system (Applied Biosystems, Foster City, CA, USA). The reaction conditions were 50 °C for 2 min following by 10 min at 95 °C and 40 cycles of 15 sec at 95 °C and 1 min at 60 °C. ΔCycle of threshold (ΔC_T_) was calculated by subtracting the C_T_ of GAPDH mRNA from the C_T_ of IL-2 or IFN-γ mRNAs.

### 3.8. Statistical analysis

Data were presented as Mean±S.D., and the differences between groups were assessed with student’s *t* test at a significant level of P < 0.05.

## 4. Conclusions

In the present study, **2-OH**, a new synthetic compound, was subjected to biological activity assay. The results indicated that **2-OH** enhanced PBMC proliferation. **2-OH** increased IL-2 and IFN-γ productions in PBMC by modulation of their gene expression. This is the first report of immunomodulatory functions on PBMC identified in **2-OH**. Cell proliferation and IL-2 and IFN-γ production of PBMC play important roles against bacterial and viral infection [18]. It suggests that **2-OH** may be an immune modulator. However, its detailed mechanisms of action are subjected for further study.
